# Kallistatin treatment attenuates lethality and organ injury in mouse models of established sepsis

**DOI:** 10.1186/s13054-015-0919-4

**Published:** 2015-05-01

**Authors:** Pengfei Li, Youming Guo, Grant Bledsoe, Zhi-Rong Yang, Hongkuan Fan, Lee Chao, Julie Chao

**Affiliations:** Department of Biochemistry and Molecular Biology, Medical University of South Carolina, 173 Ashley Ave, Charleston, SC 29425-2211 USA; Department of Neurosciences, Medical University of South Carolina, 173 Ashley Ave, Charleston, SC 29425-2211 USA

## Abstract

**Introduction:**

Kallistatin levels in the circulation are reduced in patients with sepsis and liver disease. Transgenic mice expressing kallistatin are resistant to lipopolysaccharide (LPS)-induced mortality. Here, we investigated the effect of kallistatin on survival and organ damage in mouse models of established sepsis.

**Methods:**

Mice were rendered septic by cecal ligation and puncture (CLP), or endotoxemic by LPS injection. Recombinant human kallistatin was administered intravenously six hours after CLP, or intraperitoneally four hours after LPS challenge. The effect of kallistatin treatment on organ damage was examined one day after sepsis initiation, and mouse survival was monitored for four to six days.

**Results:**

Human kallistatin was detected in mouse serum of kallistatin-treated mice. Kallistatin significantly reduced CLP-induced renal injury as well as blood urea nitrogen, serum creatinine, interleukin-6 (IL-6), and high mobility group box-1 (HMGB1) levels. In the lung, kallistatin decreased malondialdehyde levels and *HMGB1* and toll-like receptor-4 (*TLR4*) synthesis, but increased suppressor of cytokine signaling-3 (*SOCS3*) expression. Moreover, kallistatin attenuated liver injury, serum alanine transaminase (ALT) levels and hepatic tumor necrosis factor-α (*TNF-α*) synthesis. Furthermore, delayed kallistatin administration improved survival in CLP mice by 38%, and LPS-treated mice by 42%. In LPS-induced endotoxemic mice, kallistatin attenuated kidney damage in association with reduced serum creatinine, IL-6 and HMGB1 levels, and increased renal *SOCS3* expression. Kallistatin also decreased liver injury in conjunction with diminished serum ALT levels and hepatic *TNF-α* and *TLR4* expression. In cultured macrophages, kallistatin through its active site increased *SOCS3* expression, but this effect was blocked by inhibitors of tyrosine kinase, protein kinase C and extracellular signal-regulated kinase (ERK), indicating that kallistatin stimulates a tyrosine-kinase-protein kinase C-ERK signaling pathway.

**Conclusions:**

This is the first study to demonstrate that delayed human kallistatin administration is effective in attenuating multi-organ injury, inflammation and mortality in mouse models of polymicrobial infection and endotoxemia. Thus, kallistatin therapy may provide a promising approach for the treatment of sepsis in humans.

## Introduction

Sepsis is a systemic inflammatory response caused by microbial infection or bacterial products, such as lipopolysaccharide (LPS) [1]. Despite many years of dedicated research, sepsis remains one of the most frequent causes of mortality in critically ill patients, and contributes to significant economic costs in the United States [1-3]. Because numerous signaling cascades are triggered during sepsis, selective blocking of inflammatory mediators is not sufficient to arrest this process [3]. Inflammation and multi-organ dysfunction are closely associated with sepsis-induced lethality [4]. Sepsis is mediated by early (tumor necrosis factor-α (TNF-α), interleukin-6 (IL-6)) and late (high mobility group box-1 (HMGB1)) inflammatory cytokines in response to infection [1]. Although the underlying pathophysiology of sepsis has not been completely elucidated, TNF-α and HMGB1 upregulation is known to play a crucial role in the systemic inflammatory response [5-7]. Moreover, suppressor of cytokine signaling-3 (SOCS3), a feedback inhibitor of LPS-induced inflammation and immune response [8], has also been shown to be a key player in inhibiting NF-κB-mediated pro-inflammatory cytokine production and HMGB1 release [9,10]. LPS stimulates SOCS3 expression in macrophages, possibly through activation of protein kinase C (PKC), phosphoinositide 3-kinase (PI3K) and extracellular signal-regulated kinase (ERK1/2) [11,12]. Furthermore, SOCS3 deficiency was found to promote inflammation in macrophages, whereas *SOCS3* gene delivery reduced the mortality of mice with LPS-induced endotoxic shock [9,13]. Thus, approaches that broadly target the inhibition of systemic inflammation could provide effective strategies for the treatment of sepsis.

Kallistatin was first purified and characterized from human plasma as a novel serine proteinase inhibitor (serpin) and a specific tissue kallikrein inhibitor [14]. Kallistatin is an acidic glycoprotein with a pI of 4.6 and a molecular weight of 58 kDa [14,15]. As an endogenous protein, kallistatin exhibits pleiotropic effects in inhibiting inflammation, apoptosis and oxidative stress in animal models and cultured cells [16-21]. The normal plasma level of kallistatin in healthy subjects, measured by a specific ELISA, is 22.1 ± 3.5 μg/ml [22]. Circulating kallistatin levels are reduced in patients with septic syndrome and liver disease, as well as in mice with LPS-induced endotoxemia [22,23]. Transgenic mice expressing rat kallistatin display prolonged survival when subjected to endotoxic shock [23]. Moreover, kallistatin *SERPINA4* gene transfer attenuates mortality, inflammation, and liver and skin damage in mice with Gram-positive streptococcal infection [24]. Kallistatin competes with TNF-α binding to cultured endothelial cells through its heparin-binding domain, thereby antagonizing TNF-α-induced NF-κB activation, pro-inflammatory gene expression and subsequent inflammatory response [21]. Likewise, kallistatin’s heparin-binding site is essential for blocking HMGB1-induced inflammatory gene expression in endothelial cells [25]. Therefore, kallistatin is capable of inhibiting the inflammatory responses of both early (TNF-α) and late (HMGB1) cytokines. We recently reported that kallistatin pre-treatment attenuated kidney injury, inflammatory gene expression and mortality in cecal ligation and puncture (CLP)-induced septic mice [25]. In this study, we further investigated the effect and potential mechanism of delayed kallistatin administration after onset of sepsis on mortality and organ injury in mouse models of polymicrobial sepsis and endotoxic shock.

## Methods

### Purification and characterization of recombinant human kallistatin

Recombinant human kallistatin was secreted into the serum-free medium of cultured human embryonic kidney cells (HEK293T), and culture medium was concentrated by ammonium sulfate precipitation followed by nickel-affinity and heparin-affinity chromatography as previously described [25,26]. Human recombinant wild-type kallistatin, heparin-binding site mutant kallistatin and active site mutant kallistatin were purified from *Escherichia coli* as previously described [26].

### Cecal ligation and puncture-induced sepsis and survival study

CD-1 mice (male, seven to eight-weeks-old; Harlan, Indianapolis, IN, USA) were housed in a germ-free environment. All procedures complied with the standards for care and use of animal subjects as stated in the National Research Council’s *Guide for the Care and Use of Laboratory Animals*. The protocol for all animal studies was approved by the Institutional Animal Care and Use Committee at the Medical University of South Carolina (approval number: ARC#3158). All surgery was performed under anesthesia to minimize suffering. CLP was performed as described previously [25]. Briefly, the cecum was ligated at the colon juncture and then punctured twice with a 21-gauge needle. All animals were then fluid-resuscitated subcutaneously with 0.8 ml normal sterile saline. The sham operation was performed in the same way as CLP, but without ligation and puncture of the cecum. Mice were randomly assigned to one of four groups (n = 5 per group): 1) sham group receiving phosphate-buffered saline (PBS); 2) CLP control group receiving PBS; 3) CLP + KS3 group receiving 3 mg/kg body weight of kallistatin; or 4) CLP + KS10 group receiving 10 mg/kg body weight of kallistatin. Mice were injected intravenously with 0.1 ml PBS or recombinant human kallistatin in 0.1 ml PBS six hours after surgery. Mice (Harlan, Indianapolis, IN, USA) were killed 24 hours after CLP, and serum and tissues were collected for biochemical and histological analyses. For the survival study, mice were randomly assigned to one of three groups (n = 16 per group): 1) sham group receiving PBS; 2) CLP control group receiving PBS; or 3) CLP + KS20 group receiving 20 mg/kg body weight of kallistatin. At six hours after CLP surgery, mice were injected intravenously with 0.1 ml PBS or recombinant kallistatin in 0.1 ml PBS. Mouse survival was monitored every 24 hours for a total of six days.

### Lipopolysaccharide-induced endotoxemia and survival study

Endotoxemia was induced in CD-1 mice (male, seven to eight-weeks-old) by intraperitoneal injection of LPS (15 mg/kg body weight; *E. coli* LPS 0111:B4; Sigma-Aldrich, St. Louis, USA) dissolved in sterile saline. Mice were randomly assigned to one of four groups (n = 6 per group): 1) control group receiving sterile saline; 2) LPS control group receiving sterile saline; 3) LPS + KS10 group receiving 10 mg/kg body weight of kallistatin; or 4) LPS + KS20 group receiving 20 mg/kg body weight of kallistatin. At four hours after LPS injection, mice were injected intraperitoneally with 0.1 ml of sterile saline or recombinant kallistatin in 0.1 ml sterile saline. Mice were sacrificed at 22 hours after LPS challenge, and serum and tissues were collected for biochemical and histological analyses. For the survival study, mice were assigned to one of two groups (n = 16 per group): 1) LPS control group receiving sterile saline; or 2) LPS + KS20 group receiving 20 mg/kg body weight of kallistatin. Mice were injected intraperitoneally with 0.1 ml sterile saline or recombinant kallistatin in 0.1 ml sterile saline four hours after LPS injection. Mouse survival was monitored every 12 hours for a total of four days.

### Measurements of kallistatin, blood urea nitrogen, creatinine, TNF-α, IL-6, HMGB1, malondialdehyde and alanine transaminase levels in serum

Human kallistatin levels in the serum were determined by enzyme-linked immunosorbent assay (ELISA) specific for human kallistatin [22]. Blood urea nitrogen (BUN) and serum creatinine were measured with QuantiChrom Urea and Creatinine Assay Kits (BioAssay Systems, Hayward, CA, USA), respectively. Serum TNF-α levels were determined by a mouse TNF-α ELISA kit (EMD Millipore, Billerica, MA, USA). IL-6 levels in serum were measured using a mouse IL-6 ELISA kit (eBioscience, San Diego, CA, USA). Serum HMGB1 levels were determined by a mouse HMGB1 ELISA kit (Antibodies-online Inc., Atlanta, GA, USA). The levels of lung malondialdehyde (MDA) were determined as previously described [27]. Serum alanine transaminase (ALT) levels were measured using an ALT colorimetric assay kit (Cayman Chemical Company, Michigan, MI, USA).

### Tissue staining

For histological studies, kidney and liver were fixed with 4% paraformaldehyde, dehydrated, embedded and cut into 4-μm sections. Tissue sections were stained with hematoxylin and eosin (H&E) for examination of morphological damage.

### Cell culture

Mouse macrophages (RAW264.7) were cultured in Dulbecco’s modified Eagle’s medium (Gibco Invitrogen Corporation, Carlsbad, CA, USA) supplemented with heat inactivated 10% fetal bovine serum (Cellgro Mediatech Inc., Herndon, VA, USA) and 2% penicillin/streptomycin (BioWhittaker Inc., Walkersville, MD, USA) in 150 cm^2^ tissue culture flasks and maintained at 37°C in 5% CO_2_, 95% air. RAW264.7 cells within 20 passages were used for experiments. Cells were pretreated with PD98059 (ERK inhibitor, 10 μM), chelerythrine (PKC inhibitor, 1 μM) and genistein (tyrosine kinase inhibitor, 5 μM, Sigma-Aldrich, St. Louis, USA) for 30 minutes, followed by stimulation with kallistatin (0.1 μM) for 12 hours. In another set of experiments, macrophages were incubated with 0.05 μM of wild-type kallistatin, heparin-mutant kallistatin or active site mutant kallistatin in the presence of polymyxin B (10 μg/ml) for 12 hours.

### Real-time reverse transcription-polymerase chain reaction

Total RNA was extracted from lung, kidney and liver tissues and cultured macrophages with TRIzol® reagent (Invitrogen, Carlsbad, CA, USA), following the manufacturer's protocol. cDNA was synthesized with a High Capacity cDNA Reverse Transcription Kit (Applied Biosystems, Foster City, CA, USA) according to the manufacturer’s instructions. Quantitative real-time PCR was performed by Prism 7300 Real Time PCR System (Applied Biosystems) using TaqMan Gene Expression Master Mix (Applied Biosystems) in a final reaction volume of 20 μl with each primer. The following primers were purchased from Applied Biosystems: mouse *GAPDH* (Mm 99999915_gl), mouse *HMGB1* (Mm 00849805_gl), mouse *TLR4* (Mm 00445274_m1), mouse *TNF-α* (Mm 00443258_ml) and mouse *SOCS3* (Mm 00545913_s1). A negative control without cDNA did not produce any amplicons. Data were analyzed with 2^−ΔΔCt^ value calculation, using *GAPDH* for normalization.

### Western blot analysis

Macrophages were lysed with ice-cold RIPA lysis buffer (10 mM Tris, pH 7.4, 1% Triton X-100, 150 mM NaCl, 1 mM EGTA, 1 mM EDTA, 1 mM phenylmethylsulfonyl fluoride, 1 μg/ml aprotinin, 1 μg/ml leupeptin and 1 μg/ml pepstatin A, Sigma-Aldrich, St. Louis, USA). All lysed samples were kept on ice for 30 minutes, and centrifuged for 10 minutes at 4°C at 12,000 g. The supernatant was collected and stored at −20°C until further analysis. Cell lysates were subjected to 10% SDS-PAGE and transferred onto a polyvinylidene difluoride membrane. The membranes (GE Heathcare, Waukesha, WI, USA) were blocked with 7% milk in Tris-buffered saline-Tween 20 (TBST, 20 mM Tris, 500 mM NaCl, and 0.1% Tween 20) for one hour. After washing with TBST twice, membranes were incubated with primary antibody overnight at 4°C. The following primary antibodies were used: polyclonal anti-phospho-p44/42 MAPK (ERK1/2) and polyclonal anti-p44/42 MAPK (ERK1/2) (Cell Signaling, Boston, MA, USA). The membranes were washed twice with TBST and incubated with Peroxidase from horseradish (HRP) conjugated secondary antibody (Cell Signaling, Boston, MA, USA) in blocking buffer for one hour. After washing three times with TBST, immunoreactive bands were visualized by incubation with ECL plus detection reagents (GE Healthcare, Waukesha, WI, USA) for five minutes and exposure to BioMax light film (Thermo Scientific, Waltham, MA, USA). The densitometry of bands was quantified with ImageJ2 software (National Institutes of Health, Bethesda, MD, USA).

### Statistical analysis

Data are expressed as means ± standard error of the mean (SE). Statistical significance was determined by analysis of variance (ANOVA) with Fisher’s probable least-squares difference test or log-rank (Mantel-Cox) test, using GraphPad Prism software (GraphPad Software, Inc., La Jolla, CA, USA). A value of *P* <0.05 was considered statistically significant.

## Results

### Kallistatin attenuates renal injury, blood urea nitrogen, serum creatinine, IL-6 and HMGB1 levels in cecal ligation and puncture mice

**Figure 1:**
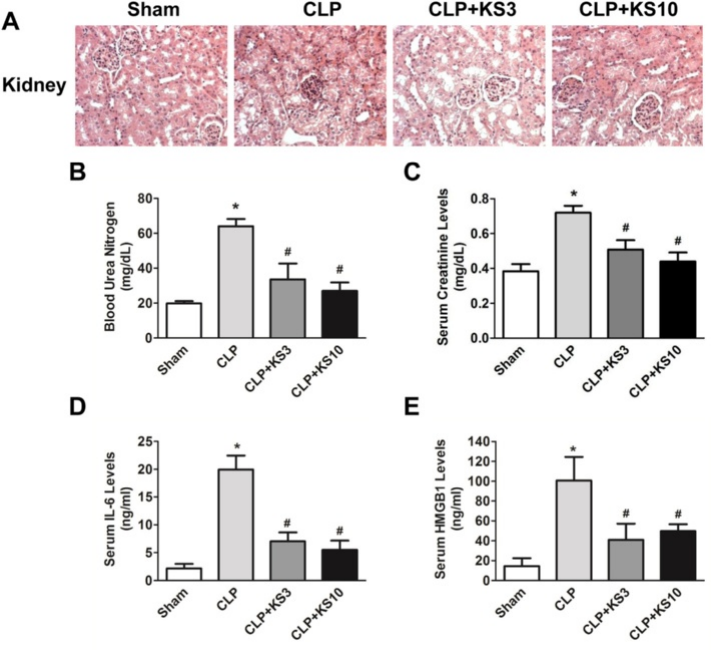
Delayed kallistatin treatment reduces renal injury and decreases blood urea nitrogen (BUN), serum creatinine, interleukin-6 (IL-6) and high mobility group box-1 (HMGB1) levels in cecal ligation and puncture (CLP) mice. **(A)** H&E staining was performed on kidney sections to examine renal histology (n = 4). The representative sections are shown at ×200 magnification. Levels of **(B)** BUN, **(C)** serum creatinine, **(D)** IL-6 and **(E)** HMGB1 were significantly decreased by kallistatin administration in CLP mice (n = 3 to 4). Data are expressed as means ± SE. *P <0.05 versus sham group; ^#^ *P* <0.05 versus CLP control group.

Human kallistatin (KS; 3 or 10 mg/kg) was injected intravenously into mice six hours after CLP surgery. Human kallistatin was detected in mouse serum at 24 hours after injection in the kallistatin-treated groups, but not in the sham or CLP control groups (CLP + KS3: 160.5 ± 26.3 ng/ml, CLP + KS10: 639.6 ± 126.7 ng/ml; n = 4). The effect of kallistatin on CLP-induced renal injury was characterized by H&E staining at 24 hours after CLP surgery (Figure 1A). Histological findings in the sham group showed renal tubules with no damage. Dilated renal tubules and swollen tubular cells were observed in the CLP control group, and slightly thickened basement membranes of renal tubules were found in CLP mice with kallistatin administration. BUN levels were markedly increased in the CLP control group, but significantly reduced by kallistatin administration (CLP control: 64.1 ± 4.2 mg/dL, CLP + KS3: 33.7 ± 8.9 mg/dL, CLP + KS10: 27.1 ± 4.8 mg/dL; n = 4, *P* <0.05; Figure 1B). Kallistatin treatment also significantly decreased CLP-induced serum creatinine levels (CLP control: 0.7 ± 0.03 mg/dL, CLP + KS3: 0.5 ± 0.05 mg/dL, CLP + KS10: 0.4 ± 0.05 mg/dL; n = 4, *P* <0.05; Figure 1C). Moreover, compared to the CLP control group, kallistatin administration reduced serum levels of IL-6 (CLP control: 19.9 ± 2.5 ng/ml, CLP + KS3: 7.0 ± 1.6 ng/ml, CLP + KS10: 5.5 ± 1.6 ng/ml; n = 3 to 4, *P* <0.05; Figure 1D) and HMGB1 (CLP control: 100.7 ± 23.7 ng/ml, CLP + KS3: 40.9 ± 16.3 ng/ml, CLP + KS10: 49.6 ± 7.0 ng/ml; n = 4, *P* <0.05; Figure 1E).

### Kallistatin decreases malondialdehyde, HMGB1 and TLR4 levels, but increases SOCS3 expression in the lungs of cecal ligation and puncture mice

**Figure 2:**
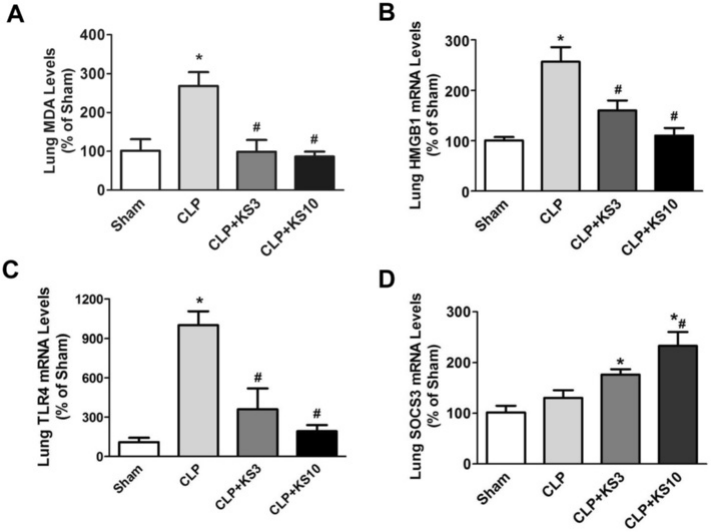
Delayed kallistatin treatment reduces malondialdehyde (MDA), high mobility group box-1 (HMGB1) and toll-like receptor-4 (TLR4) expression, and increases suppressor of cytokine signaling-3 (SOCS3) expression in lung tissue of cecal ligation and puncture (CLP) mice. Levels of **(A)** MDA, **(B)** HMGB1 and **(C)** TLR4 were significantly reduced in kallistatin-treated mice (n = 3 to 4). **(D)** Kallistatin administration also significantly increased SOCS3 expression compared to the sham group (n = 3). Data are expressed as means ± SE. *P <0.05 versus sham group; ^#^ *P* <0.05 versus CLP control group.

Kallistatin administration attenuated CLP-induced lipid peroxidation as evidenced by reduced lung MDA levels compared to the CLP control group (CLP + KS3: 63.1 ± 11.2% reduction, CLP + KS10: 67.8 ± 4.8% reduction; n = 4, *P* <0.05; Figure 2A) Lung *HMGB1* expression was increased in the CLP control group, but significantly reduced by kallistatin treatment (CLP + KS3: 37.6 ± 7.7% reduction, CLP + KS10: 57.0 ± 5.9% reduction; n = 3, *P* <0.05; Figure 2B). Kallistatin treatment also markedly decreased CLP-induced lung *TLR4* expression (CLP + KS3: 63.9 ± 15.9% reduction, CLP + KS10: 80.9 ± 4.6% reduction; n = 3, *P* <0.05; Figure 2C). Moreover, mice receiving kallistatin treatment at the lower dose (KS3) exhibited a significant increase in lung *SOCS3* expression compared with the sham group (CLP + KS3: 1.7 ± 0.1 fold; n = 3, *P* <0.05; Figure 2D), whereas kallistatin administration at the higher dose (KS10) further increased lung *SOCS3* expression compared to the CLP control group (CLP + KS10: 1.8 ± 0.2 fold; n = 3, *P* <0.05; Figure 2D).

### Kallistatin reduces liver injury, serum alanine transaminase levels and hepatic TNF-α expression in cecal ligation and puncture mice

**Figure 3:**
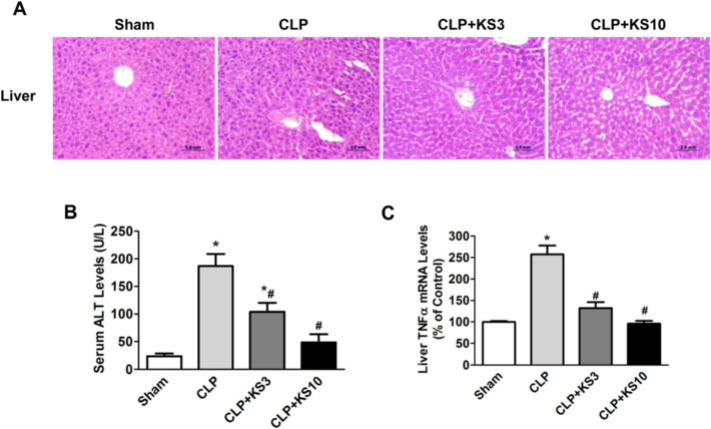
Delayed kallistatin administration attenuated liver injury, serum alanine transaminase (ALT) levels and liver tumor necrosis factor-α (TNF-α) expression in cecal ligation and puncture (CLP) mice. **(A)** H&E staining was performed on liver sections to examine liver histology (n = 4). Representative sections are shown at ×200 magnification. **(B)** Serum ALT levels and **(C)** liver TNF-α expression were significantly decreased in kallistatin-treated CLP mice (n = 3 to 4). Data are expressed as means ± SE. *P <0.05 versus sham group; ^#^ *P* <0.05 versus CLP control group.

The effect of kallistatin on liver injury was evaluated by H&E staining at 24 hours after polymicrobial infection. As shown in Figure 3A, liver tissues from the CLP control group showed swollen hepatocytes and red blood cell infiltration, but the effects were reversed after kallistatin treatment. Kallistatin administration decreased CLP-induced serum ALT levels (CLP control: 186.8 ± 22.0 U/L, CLP + KS3: 101.1 ± 16.2 U/L, CLP + KS10: 48.5 ± 14.7 U/L; n = 3 to 4, *P* <0.05; Figure 3B). Kallistatin administration also significantly reduced CLP-induced hepatic *TNF-α* expression (CLP + KS3: 49 ± 5.4% reduction, CLP + KS10: 63 ± 2.5% reduction; n = 3, *P* <0.05; Figure 3C) compared to the CLP control group.

### Delayed kallistatin administration prolongs survival of polymicrobial septic and endotoxemic mice

**Figure 4:**
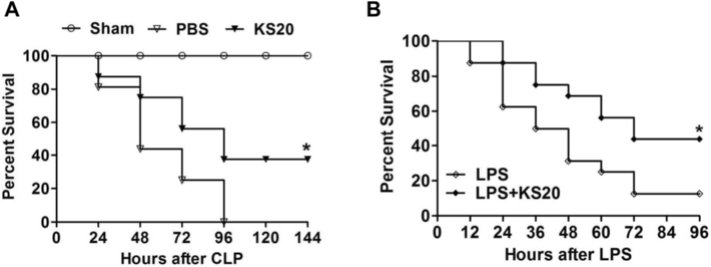
Delayed kallistatin treatment improves survival in cecal ligation and puncture (CLP) mice and lipopolysaccharide (LPS)-induced endotoxemic mice. **(A)** Mice receiving human kallistatin (20 mg/kg, KS20) presented significantly reduced mortality during the observation period compared to CLP control mice (PBS group); sham-operated mice exhibited 100% survival (n = 16). **(B)** Mice receiving human kallistatin (20 mg/kg, KS20) exhibited significantly reduced mortality rate during the observation period compared to LPS control mice (n = 16). *P <0.05 versus CLP control group or LPS control group.

To determine the effect of kallistatin on CLP-induced mortality, kallistatin (20 mg/kg) was injected intravenously into mice at six hours after CLP surgery. A mortality study was conducted and the survival of CLP mice was monitored every 24 hours for a total of six days. Sham-operated mice (n = 16) exhibited 100% survival. All CLP control mice died within four days after CLP, whereas kallistatin administration (20 mg/kg) increased the survival rate by 38% compared to CLP control mice (n = 16, *P* <0.05; Figure 4A). To determine the effect of kallistatin on LPS-induced mortality, sterile saline or kallistatin (20 mg/kg) was injected intraperitoneally into mice four hours after LPS injection (15 mg/kg). Mouse survival was monitored every 12 hours for a total of four days. Treatment with kallistatin significantly improved the survival rate by 42% compared to the LPS control mice (n = 16, *P* <0.05; Figure 4B).

### Kallistatin reduces renal injury and serum creatinine, TNF-α, IL-6 and HMGB1 levels, but increases renal SOCS3 expression in endotoxemic mice

**Figure 5:**
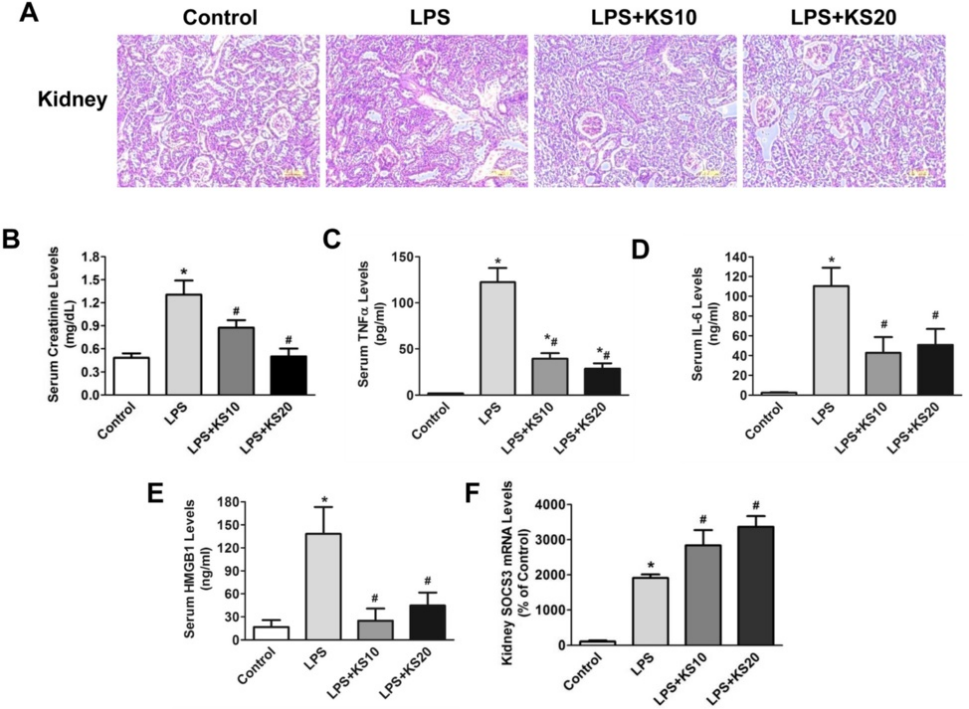
Delayed kallistatin treatment reduces renal injury, serum creatinine, tumor necrosis factor-α (TNF-α), interleukin-6 (IL-6) and high mobility group box-1 (HMGB1) levels, and increases renal suppressor of cytokine signaling-3 (SOCS3) expression in endotoxemic mice. **(A)** H&E staining was performed on kidney sections to examine histology (n = 4). The representative sections are shown at ×200 magnification. **(B)** Serum creatinine levels were attenuated by kallistatin treatment. **(C)** TNF-α, **(D)** IL-6 and **(E)** HMGB1 levels in serum of kallistatin-treated mice were significantly lower than that of the lipopolysaccharide (LPS) control mice. **(F)** Kallistatin treatment also significantly increased renal SOCS3 expression (n = 3 to 6). Data are expressed as means ± SE. *P <0.05 versus control group; ^#^ *P* <0.05 versus LPS control group.

Human kallistatin levels were detected in mouse serum at 22 hours after LPS injection in the kallistatin-treated groups (10 or 20 mg/kg), but not in the control or LPS control groups (LPS + KS10: 1377 ± 141.5 ng/ml, LPS + KS20: 2715 ± 176.3 ng/ml; n = 4 to 5). H&E staining was performed to evaluate the effect of kallistatin on LPS-induced kidney injury at 22 hours after LPS injection. As shown in Figure 5A, treatment with kallistatin ameliorated brush border loss, decreased the number of infiltrated immune cells and reduced tubular injury in the kidneys of mice injected with LPS. Kallistatin treatment significantly lowered LPS-induced serum creatinine levels (LPS control: 1.3 ± 0.2 mg/dL, LPS + KS10: 0.8 ± 0.1 mg/dL, LPS + KS20: 0.5 ± 0.1 mg/dL; n = 4 to 6, *P* <0.05; Figure 5B). Compared to the LPS control group, kallistatin administration markedly reduced serum levels of TNF-α (LPS control: 122.4 ± 15.3 pg/ml, LPS + KS10: 39.6 ± 5.8 pg/ml, LPS + KS20: 28.7 ± 5.9 pg/ml; n = 3 to 4, *P* <0.05; Figure 5C) and IL-6 (LPS control: 110.3 ± 18.7 ng/ml, LPS + KS10: 42.5 ± 16.1 ng/ml, LPS + KS20: 50.7 ± 16.1 ng/ml; n = 4 to 5, *P* <0.05; Figure 5D). Moreover, kallistatin treatment reduced LPS-induced elevation of serum HMGB1 levels (LPS control: 138 ± 35.1 ng/ml, LPS + KS10: 24.9 ± 15.9 ng/ml, LPS + KS20: 44.6 ± 17 ng/ml; n = 4, *P* <0.05; Figure 5E). LPS challenge dramatically upregulated renal *SOCS3* expression compared to the control group (LPS control: 18.1 ± 0.9 fold; n = 3, *P* <0.05; Figure 5F), and kallistatin administration further increased renal *SOCS3* expression compared to the LPS control group (LPS + KS10: 1.5 ± 0.2 fold, LPS + KS20: 1.8 ± 0.2 fold; n = 3, *P* <0.05; Figure 5F).

### Kallistatin reduces liver injury, serum alanine transaminase levels and TNF-α and TLR4 expression in endotoxemic mice

**Figure 6:**
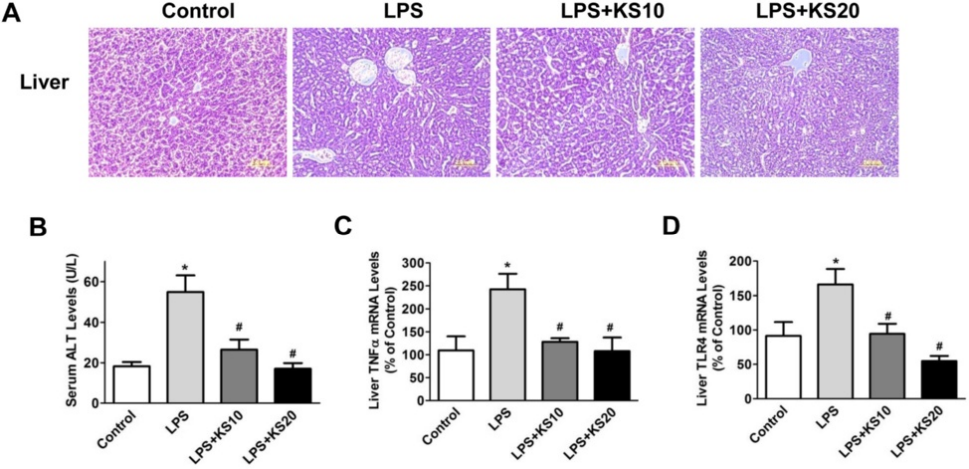
Kallistatin treatment ameliorates liver injury, attenuates serum alanine transaminase (ALT) levels, and reduces tumor necrosis factor-α (TNF-α) and toll-like receptor-4 (TLR4) expression in liver tissue of LPS-induced endotoxemic mice. **(A)** H&E staining was performed on liver sections to examine histology (n = 4). The representative sections are shown at ×200 magnification. **(B)** Kallistatin administration reduces serum ALT levels (n = 3 to 4). Liver mRNA levels of **(C)** TNF-α and **(D)** TLR4 were significantly decreased by kallistatin treatment (n = 3). *P <0.05 versus control group; ^#^ *P* <0.05 versus LPS control group.

H&E staining was performed to evaluate the effect of kallistatin on LPS-induced liver injury at 22 hours after LPS injection. As shown in Figure 6A, liver tissues from the LPS control group contained cracks, fibrin deposits and infiltrated red blood cells that were less frequent in kallistatin-treated groups. Kallistatin treatment attenuated LPS-induced serum ALT levels (LPS control: 54.9 ± 8.2 U/L, LPS + KS10: 26.5 ± 5.0 U/L, LPS + KS20: 17.8 ± 2.8 U/L; n = 3 to 4, *P* <0.05; Figure 6B). Kallistatin administration also attenuated LPS-induced hepatic *TNF-α* (LPS + KS10: 47 ± 3.2% reduction, LPS + KS20: 56 ± 15.1% reduction; n = 3, *P* <0.05; Figure 6C) and *TLR4* expression (LPS + KS10: 43 ± 8.9% reduction, LPS + KS20: 67 ± 4.5% reduction; n = 3, *P* <0.05; Figure 6D) compared to the LPS control group.

### Kallistatin via its active site induces SOCS3 expression through tyrosine kinase-PKC-ERK signaling in cultured macrophages

**Figure 7:**
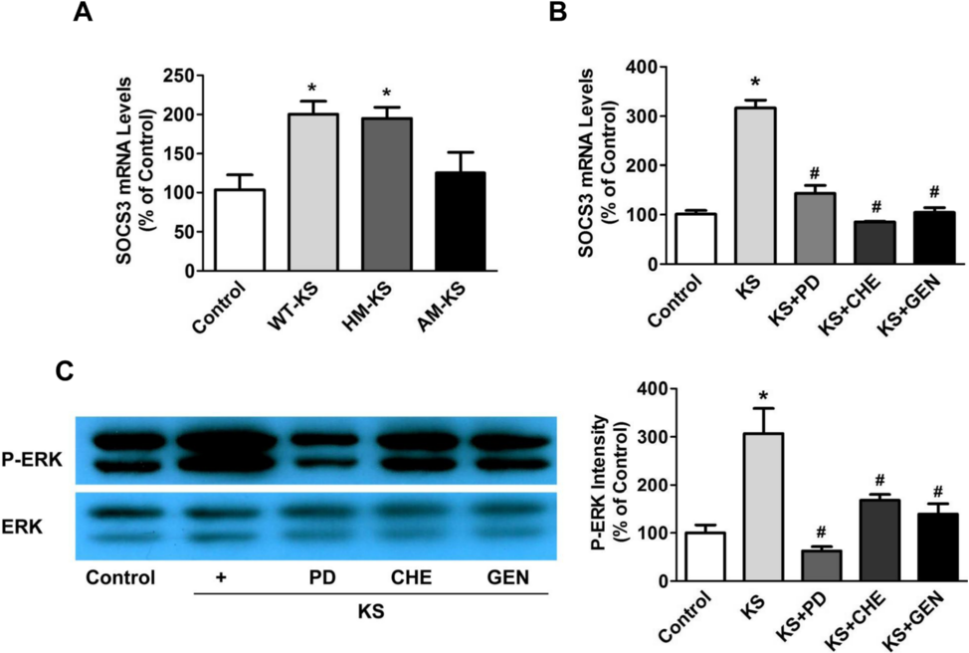
Kallistatin treatment enhances suppressor of cytokine signaling-3 (SOCS3) expression through activation of a tyrosine kinase-protein kinase C (PKC)-extracellular signal-regulated kinase (ERK) signaling pathway in RAW264.7 cells. **(A)** Macrophages were incubated with 0.05 μM wild-type kallistatin (WT-KS), heparin-mutant kallistatin (HM-KS) or active site-mutant kallistatin (AM-KS) for 12 hours. Wild-type or heparin mutant kallistatin, but not active site mutant kallistatin, significantly increased SOCS3 expression (n = 3). **(B)** The effect of kallistatin on SOCS3 expression in macrophages was blocked by PD98059 (PD; ERK inhibitor), chelerythrine (CHE; PKC inhibitor) and genistein (GEN; tyrosine kinase inhibitor). **(C)** ERK phosphorylation induced by kallistatin in macrophages was also reversed by PD98059, chelerythrine and genistein (n = 3). *P <0.05 versus control group; ^#^ *P* <0.05 versus kallistatin alone.

Kallistatin contains two important structural elements: an active site and a heparin-binding domain [28,29]. Kallistatin, via its heparin-binding site, has been shown to inhibit TNF-α- and HMGB1-mediated inflammation in cultured endothelial cells [21,25]. We therefore examined the role of kallistatin’s structural elements in kallistatin-induced *SOCS3* expression in cultured macrophages. *SOCS3* expression was significantly increased by wild-type kallistatin (1.9 ± 0.2 fold, n = 3, *P* <0.05) and heparin-binding site mutant kallistatin (1.9 ± 0.1 fold, n = 3, *P* <0.05), but not active site mutant kallistatin (n = 3, *P* >0.05; Figure 7A). These results suggest that kallistatin, via its active site, induces *SOCS3* expression. Moreover, kallistatin markedly induced *SOCS3* expression more than three-fold (KS: 3.1 ± 0.2 fold; n = 3, *P* <0.05), and the effect was blocked by PD98059 (ERK inhibitor, 10 μM; 55 ± 5.2% reduction; n = 3, *P* <0.05), chelerythrine (PKC inhibitor, 1 μM; 73 ± 0.5% reduction; n = 3, *P* <0.05) and genistein (tyrosine kinase inhibitor, 5 μM; 67 ± 3.0% reduction; n = 3, *P* <0.05; Figure 7B). However, kallistatin’s effect on *SOCS3* expression was not affected by LY294002 (PI3K inhibitor) or L-NAME (nitric oxide synthase (NOS) inhibitor), indicating an event unrelated to the PI3K-Akt-NOS pathway (data not shown). In addition, kallistatin significantly increased ERK1/2 phosphorylation (3.1 ± 0.5 fold, n = 3, *P* <0.05), which was reduced by PD98059 (80 ± 3.0% reduction; n = 3, *P* <0.05), chelerythrine (45 ± 4.0% reduction; n = 3, *P* <0.05) and genistein (55 ± 7.0% reduction; n = 3, *P* <0.05; Figure 7C). These results indicate that kallistatin induces *SOCS3* expression via activating tyrosine kinase-PKC-ERK signaling.

## Discussion

This is the first study to demonstrate that kallistatin administration after onset of sepsis protects against lethality and multi-organ damage in mouse models of both polymicrobial infection and LPS-induced endotoxemia. Human kallistatin not only improved the survival of mice with established sepsis, but also significantly reduced organ injury in the kidney, lung and liver. Kallistatin treatment markedly lowered systemic inflammation as indicated by decreased circulating levels of the pro-inflammatory cytokines TNF-α, IL-6 and HMGB1. Our findings also indicate that kallistatin dramatically upregulated *SOCS3* expression, a negative regulator of inflammation, in the lung and kidney. Moreover, kallistatin’s active site plays a crucial role in inducing *SOCS3* expression via activation of a tyrosine kinase-PKC-ERK pathway in cultured macrophages. Our previous studies indicated that kallistatin, through its heparin-binding site, blocks TNF-α and HMGB1-induced inflammatory responses [21,25]. Collectively, these findings reveal a protective role and unique mechanisms of kallistatin in mortality, systemic inflammation and multi-organ damage in mice with established sepsis. Importantly, as sepsis is the second leading cause of death in non-coronary ICU patients [30], kallistatin may be a novel strategy for combating sepsis syndrome in humans.

Severe sepsis is characterized by systemic inflammation, organ damage and a high rate of mortality. The crucial role of inflammatory cytokines in the pathogenesis of sepsis has been convincingly demonstrated [7]. Numerous cytokines and inflammatory mediators, including IL-1, IL-6, IL-12 and TNF-α, are documented to be released in the early phase of sepsis, while HMGB1 is released at the late phase [7]. High serum concentrations of TNF-α are correlated with sepsis-induced mortality [31]. It has also been shown that blockade of IL-6 and HMGB1 increases the survival of septic animals with established sepsis [32,33]. Our present study shows that delayed administration of human kallistatin markedly attenuated kidney, liver and lung injury in association with reduced serum levels of TNF-α, IL-6 and/or HMGB1 in mice with established sepsis. Kallistatin, through its heparin-binding site, inhibits TNF-α- and HMGB1-induced inflammation by antagonizing their binding to endothelial cell surfaces [21,25]. Therefore, kallistatin can effectively lower both early and late phase systemic inflammatory cytokine levels and their downstream effects during sepsis. This suggests that the beneficial effect of kallistatin on sepsis-induced organ injury and mortality is mainly attributed to its inhibition of inflammatory cytokine expression.

**Figure 8:**
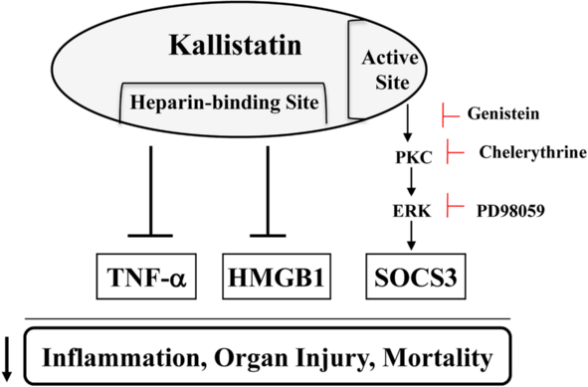
Proposed mechanisms mediated by kallistatin in sepsis-induced inflammation, organ injury and mortality. Kallistatin, via its heparin-binding domain, antagonizes TNF-α- and HMGB1-mediated inflammatory gene expression, and its active site is essential for inducing SOCS3 expression. HMGB1, high mobility group box-1; SOCS3, suppressor of cytokine signaling-3; TNF-α, tumor necrosis factor-α.

SOCS3 plays an important role in protection against sepsis as it is a negative regulator of LPS-induced inflammation [34]. A previous report showed that kallistatin protects against LPS-induced inflammation and TNF-α production via upregulation of macrophage SOCS3 expression [35]. Our current study also indicates that kallistatin attenuated inflammation and organ injury in association with increased SOCS3 expression in the lung and kidney of mice with polymicrobial infection or LPS-induced endotoxic shock. LPS and inflammatory cytokines were shown to induce SOCS3 expression via PKC-ERK or PI3K-ERK signaling [11]. Our results indicate that kallistatin increased *SOCS3* expression in cultured macrophages via activation of a tyrosine kinase-PKC-ERK signaling pathway, as kallistatin’s effects on ERK phosphorylation and *SOCS3* expression were blocked by inhibitors of tyrosine kinase, PKC and ERK1/2. Kallistatin’s effect on SOCS3 expression was not affected by LY294002 or L-NAME, indicating an event independent of the PI3K-Akt-NOS pathway (data not shown). Kallistatin contains two structural elements: an active site and a heparin-binding domain [28,29]. Kallistatin, via its heparin-binding site, interacts with cell surface heparan sulfate, thus blocking the binding of TNF-α or HMGB1 to their receptors and subsequent inflammatory responses [21,25]. In the present study, we showed that kallistatin, through its active site, stimulated *SOCS3* expression by activating a cell surface tyrosine kinase, and thus PKC-ERK signaling. Therefore, kallistatin protects against sepsis-induced inflammation, organ damage and mortality by antagonizing TNF-α- and HMGB1-mediated inflammatory gene expression, and by inducing *SOCS3* synthesis (Figure 8). Kallistatin was shown to increase endothelial NOS expression and activation, and thus NO formation, leading to inhibition of vascular inflammation [20,36]. Taken together, these findings indicate that kallistatin is a unique anti-inflammatory agent by modulating differential signaling pathways.

LPS stimulates expression of SOCS3, which in turn negatively regulates LPS-induced TLR signaling [8,34]. TLRs elicit systemic inflammatory responses and contribute to organ injury and high mortality in animal models of sepsis [37]. TLR2 and TLR4 expression are increased in septic patients, and inhibition of TLR signaling and expression was shown to improve the survival of septic mice [38]. TLR4 is considered to be the main receptor mediating the inflammatory activity of HMGB1 [39]. Kallistatin pre-treatment was found to reduce *TLR4* expression in the lungs and kidneys of CLP mice [25]. The present study also shows that delayed kallistatin administration decreased sepsis-induced *TLR4* expression in the lung and liver. Therefore, kallistatin most likely modulates TLR signaling and expression through induction of SOCS3 synthesis, leading to protection against sepsis-induced inflammatory response and organ injury.

Dysfunction of the kidney, lung and liver are common morbidities associated with sepsis [40]. Interestingly, the kallistatin gene (*SERPINA4*) is strongly correlated with a decreased risk of developing acute kidney injury in patients with septic shock [41]. Herein, we show that administration of kallistatin after onset of sepsis improved kidney and liver dysfunction in mouse models with polymicrobial infection and LPS injection. Moreover, delayed kallistatin treatment suppressed oxidative stress in the lung as indicated by reduced MDA levels in CLP mice. Oxidative stress activates inflammatory pathways and triggers a series of events that lead to organ injury [42]. Thus, these findings indicate that kallistatin protects against sepsis-induced organ injury through reduction of oxidative stress and inflammation. In addition to preventing established sepsis-induced organ injury, kallistatin gene delivery or kallistatin protein treatment has been shown to improve survival in various animal models [23-25,35]. Transgenic mice overexpressing kallistatin were more resistant to Gram-negative *E. coli* LPS-induced mortality [23]. Moreover, kallistatin gene transfer increased survival and reduced liver and skin injury, as well as inflammation, in mice with Gram-positive streptococcal infection [24]. Furthermore, kallistatin (kallikrein-binding protein) pre-treatment at two hours before LPS injection was observed to improve the survival of endotoxemic mice [35]. Likewise, we recently reported that kallistatin pre-treatment reduced mortality in septic mice after CLP surgery [25]. Importantly, the present study demonstrates that delayed administration after onset of sepsis significantly improves the survival of mice with polymicrobial infection and endotoxemia. Therefore, kallistatin could be a promising new agent for the treatment of sepsis.

## Conclusions

This study demonstrated that delayed human kallistatin treatment after onset of sepsis reduces inflammation, organ injury and mortality in both septic mouse models with polymicrobial infection and endotoxic shock. Moreover, this is the first study to indicate that kallistatin’s active site plays a key role in stimulating *SOCS3* expression via tyrosine kinase-PKC-ERK signaling in immune cells. As kallistatin is an endogenous molecule, kallistatin therapy may lead to the protection and/or reversal of sepsis-induced injury. The unique anti-inflammatory actions of kallistatin need to be further explored as a novel therapeutic approach for sepsis.

## Key messages

- Delayed administration of human kallistatin reduced organ damage, inflammation and lethality in mouse models of polymicrobial infection and lipopolysaccharide-induced endotoxemia.
- Kallistatin treatment attenuated established sepsis-induced inflammation and multi-organ injury in association with reduced *TNF-α* and *HMGB1* expression, as well as increased *SOCS3* synthesis.
- Kallistatin’s active site is essential for inducing *SOCS3* expression by activation of tyrosine kinase-PKC-ERK signaling.
- Kallistatin is a unique anti-inflammatory agent with pleiotropic actions, leading to significant protection against established sepsis.

## Abbreviations

ALT: alanine transaminase
AM-KS: active site-mutant kallistatin
BUN: blood urea nitrogen
CLP: cecal ligation and puncture
ERK: extracellular signal-regulated kinase
HMGB1: high mobility group box-1
HM-KS: heparin-mutant kallistatin
IL: interleukin
LPS: lipopolysaccharide
MDA: malondialdehyde
PKC: protein kinase C
SOCS3: suppressor of cytokine signaling-3
TLR: toll-like receptor
TNF: tumor necrosis factor
WT-KS: wild-type kallistatin
